# Granular Conjunctivitis with and without Pannus

**Published:** 1887-05

**Authors:** W. Cheatham

**Affiliations:** Lecturer on Diseases of Eye, Ear, Throat and Nose, University of Louisville; Attending Eye, Ear, Throat and Nose Physician to Louisville City Hospital; Masonic Widows and Orphans’ Home and Infirmary, Louisville, Ky.


### Clinic Reports.

### GRANULAR CONJUNCTIVITIS WITH AND WITHOUT PANNUS.

BY W. CHEATHAM, M. D.,

LECTURER ON DISEASES OF EYE, EAR, THROAT AND NOSE, UNIVERSITY OF LOUISVILLE; ATTENDING EYE, EAR, THROAT AND NOSE PHYSICIAN TO LOUISVILLE CITY HOSPITAL; MASONIC WIDOWS AND ORPHANS' HOME AND INFIRMARY, LOUISVILLE, KY.

In the few words to follow, I wish to speak especially of the management of that form of granular conjunctivitis known as trachoma, and not to the form in which we have simple hypertrophy of papilla. I shall suppose that all know the differential diagnosis, and report a few cases, calling attention mainly to their management. Granular conjunctivitis originates from bad hygiene, contagion, straining eyes, mistreated cases of catarrhal conjunctivitis, suppurative conjunctivitis, etc. Cases originating in crowded institutions, especially where many children are crowded together, should be isolated for weeks and months, or until the last symptom of the disease has disappeared. Each child in such institutions should have its own basin, towels and handkerchiefs. No child should be permitted to enter such institutions without a thorough examination for conjunctival diseases. In the Kentucky Feeble Minded Institute, of Frankfort, Ky., over forty cases of obstinate and in many of the cases damaging conjunctivitis originated from the introduction of one case. I had nearly a similar experience from the same case in one of the orphan asylums of this city. I have been months getting rid of the seed sown by this one case. To illustrate the carelessness of physicians who should know better, I had a case in point only four weeks ago. A gentleman wishing a nurse for his four children applied to me at one of our institutions. A girl fourteen years old was given him. He noticed her eyes were inflamed, but the oculist of the institution said there was no danger. He brought her to me, and I found her with a most pronounced case of granular conjunctivitis with pannus. Under my advice she was returned to the asylum. It is true the hygienic surroundings of the children she was to nurse were far better than those in the asylum, which decreased the dangers many times, yet the chances for inoculation were excellent, and who could have foretold the result? When cases of simple granular conjunctivitis come to me, I put the eyes immediately under the influence of atropia sulph. for the double purpose of putting the eyes at rest and testing them for errors of refraction. Errors of refraction, near-sightedness, over-sightedness and astigmatism are often at the bottom of the trouble, and unless corrected will make the case very much more obstinate, and cause frequent relapses. In these relapsing cases, the nose should be looked into well, as frequently the cause will be discovered there. The hygienic surroundings should be inquired into closely, and when at fault corrected. All sweets and tobacco, and in children tea and coffee, should be forbidden; all dusty, smoky places avoided, also bright lights and heat. All excesses must be corrected. After attending to this, the atropia is continued as long as any good results can be gotten. For home use I give them an eye-bath of simple salt water, or dest. ext. hamamelis, one teaspoonful to three or four of warm or cold water, to be left to the discretion of the patient. To prevent any disagreeable local effect from the continued use of the atropia—as this often produces conjunctival inflammation—I give a drop, such as the following, to be used two or three times a day: ℞. Soda bicarbonate, acid boracic aa gr. v in either aqua camph., rose water ℥℥i or hamamelis ʒss. and aqua dest. ʒiiss, or zinc sulph. grss may be added. Direct patient to lie on back, and put two or three drops in inner canthus, lift lids with fingers and let the medicine get well into the *cul-de-sacs*. When possible I see them at my office three times a week, and treat them with either argent. nit. gr. v or gr x to aqua des. ℥i. applied with brush and washed off immediately with salt water; or I apply ammonia muriate, cupri sulph. or alum in substance to everted lid. For office use now, and for many cases at home, especially when there is little or no discharge or suppuration, I use the following ointment, turning the lid and rubbing it well into the conjunctiva: ℞. Hyd. oxid. flav. grs. i—ii.—iii.—iv. or grs. vi. cocaine muriate gr. vi. lanolin and olive oil aa. ʒ ii. This is to be put in once or twice a day, but not too close to bed-time. The ointment should be rubbed thoroughly, or else free crystals of hyd. oxid. flav. will be left and do harm. The olive oil is added to soften the salve, as lanolin is very firm. There are apparently great differences in the quality of lanolin; some appears to be more acid than the others, and causes more pain. The treatment in many of these cases lasts for weeks and months, and changes will have to be made often, as the effect of first one, then the other *wears out.* Now in those cases where the blood-vessels run over the cornea, producing pannus, cases that have hung on for a long time and passed through many different hands, I say, in these cases I want to put in a word for jequirity; and again, I suppose all know by this time what jequirity is. I use nothing but the impalpable powder—powder that has been bolted several times, without a particle of grit in it, and prepared by Mr. J. Flesner, of this city. Why the powder? For several reasons. Its action can be confined better; it can be kept forever, the solutions decomposing easily. One application always answers. While advising the use of jequirity, I am aware of what has been written against it by Dr. Knapp and others. I am aware of the character and the severity of inflammation it produces, and yet I use it frequently with the happiest results, and often after having used it with a patient once, I give them some of the powder to take home and use it themselves if it becomes necessary. I have used it in acute cases, where there was free suppuration, or rather chronic cases with acute exacerbations, where all other treatment had failed, with nothing but the happiest result. How many times I have used it I would not undertake to say, and without an accident. The inflammation it produces is very severe, or else we would get no good. The beauty of it, though, outside of the wonderful effect it has in getting rid of the granulations and pannus, is the ease with which the inflammation it produces can be controlled. I use the jequerrity as follows: I make a mop by twisting a little cotton on a stick, or sometimes use a camel's-hair brush, dipping it into the powder dry, everting the lid and applying the powder over the part I wish to treat. As I said a moment ago, you can in many cases confine its action to certain parts of the conjunctiva. The lid is put back into position, and the patients given the following directions: the eye is not to be even washed unless the pain and swelling is very great. If the pain should be very great, the patient must stand it for an hour or so to allow the medicine to get in its work. After an hour or so, cold or iced cloths can be applied, and the eye bathed in a weak solution of either boric or carbolic acid. This, in a large majority of the cases, controls the inflammation and pain very readily. In a few cases this does not stop the pain; then I direct that the same opiate be given, and next morning the patient finds all pain gone. Suppose in an occasional case we should get an ulcer or slough of cornea, such occasionally follows extraction of cataract, and yet we still extract them. These eyes with pannus are often as useless as eyes with cataract, and jequerrity in the former gives as good results often as extraction in the latter.

I do not believe that one or two unfortunate results in the use of any one medicine, or following any operation, should condemn either. Some have said it should be used only in those cases where the conjunctiva is atrophied. In such cases it will produce no inflammation scarcely. I say try all the usual treatment first, and if it fails use the jequerrity as I have directed, and I feel sure it will not disappoint you. I cannot, of course, say it will give success in all cases; in some jequerrity even fails. For a few illustrative cases I will draw on my case-book:

Mr. J., aged 45. Had been treated in Cincinnati, and by myself, besides having run the gamut of all the local physicians of his town. His vision was only perception of light. He had been in this condition for several months. Lids badly granulated and cornea a mass of blood-vessels. No view of iris could be gotten. The powder was put in about 11 o'clock a. m. At 4 p. m., same day, I was sent for, as he was suffering greatly. The lids were so œdematous that no view of the globe could be gotten. I never saw more severe reaction. Cold carbolized applications and a quarter of a grain of morphia soon had him comfortable. In six days Mr. J. walked to my office and returned home on the railroad alone. Three years afterwards he returned with a relapse, and was again similarly treated with similar result. The relapse was brought on by working in tobacco.

Mrs. R., of Indiana, in almost as bad a condition, recovered vision enough in two weeks to read.

Miss W., of Tennessee, an anæmic young lady, who had been shut up in a dark room for months, a most unpromising case, after two applications, two weeks apart, was returned home with excellent vision. These are a few of the many. The most promising cases are those that might be called acute pannus.

I put in this plea for jequerrity, hoping it will prevent a good drug, when properly used, being thrown aside as a dangerous one. As I have stated before, I have used it numerous times myself, sent it to other doctors to use who had never seen it before, given it to my patients on whom I had used it once to take home and use themselves, and they have used it on their neighbors; one man especially used it on four members of his family, and without an accident.

